# Flight is the key to postprandial blood glucose balance in the fruit bats *Eonycteris spelaea* and *Cynopterus sphinx*

**DOI:** 10.1002/ece3.3416

**Published:** 2017-09-19

**Authors:** Xingwen Peng, Xiangyang He, Qi Liu, Yunxiao Sun, Hui Liu, Qin Zhang, Jie Liang, Zhen Peng, Zhixiao Liu, Libiao Zhang

**Affiliations:** ^1^ Guangdong Key Laboratory of Animal Conservation and Resource Utilization Guangdong Public Laboratory of Wild Animal Conservation and Utilization Guangdong Institute of Applied Biological Resources Guangzhou China; ^2^ College of Biology and Environmental Sciences Jishou University Jishou China

**Keywords:** Chiroptera, flight, frugivorous, glucose metabolism, nectarivorous

## Abstract

Excessive sugar consumption could lead to high blood glucose levels that are harmful to mammalian health and life. Despite consuming large amounts of sugar‐rich food, fruit bats have a longer lifespan, raising the question of how these bats overcome potential hyperglycemia. We investigated the change of blood glucose level in nectar‐feeding bats (*Eonycteris spelaea*) and fruit‐eating bats (*Cynopterus sphinx*) via adjusting their sugar intake and time of flight. We found that the maximum blood glucose level of *C. sphinx* was higher than 24 mmol/L that is considered to be pathological in other mammals. After *C. sphinx* bats spent approximately 75% of their time to fly, their blood glucose levels dropped markedly, and the blood glucose of *E. spelaea* fell to the fast levels after they spent 70% time of fly. Thus, the level of blood glucose elevated with the quantity of sugar intake but declined with the time of flight. Our results indicate that high‐intensive flight is a key regulator for blood glucose homeostasis during foraging. High‐intensive flight may confer benefits to the fruit bats in foraging success and behavioral interactions and increases the efficiency of pollen and seed disposal mediated by bats.

## 1 INTRODUCTION

Flight is the most economical patterns of animal location according to the energy consumption per unit of distance travelled. However, flapping during flight is an inefficient means of movement because it consumes more energy per unit of time than any other mode of locomotion (Alexander, 2002; Masman & Klaassen, 1987; Schmidt‐Nielsen, 1997). In vertebrates, the rate of flight metabolism is eightfold to 15‐fold higher than that of basal metabolism (Speakman & Thomas, 2003) and the energy consumption during flight is 10‐ to 12‐fold higher than that during rest (Voigt & Winter, 1999). These are also metabolic patterns for nectar‐feeding bats (Phyllostomidae: Glossophaginae).

Animals carefully choose their fuel resource during flight because flight demands significantly higher energy expenditure (Hambly, Harper, & Speakman, 2004). In general, they select the fuel resource according to their nutritional and activity status (Amitai et al., 2010). Chiroptera, commonly called bats, are the second largest order of mammals, with relatively high species diversity and various feeding habits, and they are also the only mammals that can fly. Among Chiroptera, both nectarivorous and frugivorous fruit bats are known to have energy‐rich foods (e.g., nectar and fruits) and to absorb carbohydrate‐rich foods relatively fast and efficient (Kelm, Simon, Kuhlow, Voigt, & Ristow, 2011). The sugar content of some fruits and nectar can be 30%, or even higher (Baker, Baker, & Hodges, 1998). Fruit bats uptake large amounts of sugar in daily diet; however, whether they overcome potential hyperglycemia after feeding is not known. Fruit bats, such as Glossophagine bat, a small bat (approximately 10 g) in the family Phyllostomidae may consume nearly a 1.5‐fold of its body mass in nectar or a quarter of its body mass in sugar each night (Helversen & Reyer, 1984; Kelm et al., 2008; Voigt, Kelm, & Visser, 2006). Despite ingesting such high amounts of sugar, fruit bats have a longer lifespan compared with those nonflying homeotherms of a similar body weight (Brunet‐Rossinni & Austad, 2004; de Magalhães, Costa, & Church, 2007).

Considering the high costs of searching food by flying, bats may tend to consume more nutriment to supply the energy needs during foraging. In nature, however, unlike insectivorous bats (Grinevitch, Holroyd, & Barclay, 1995; Jones & Rydell, 1994), fruit bats feed intermittently over a time period of 12 hr (Winter & Von Helversen, 1998), which is a comparatively long time span. Thus, it is conceivable that fruit bats are well‐equipped with intestinal sucrase, a digestive enzyme that enables a rapid absorption of ingested sugar (Hernandez & del Rio, 1992). Both flying and resting fruit bats can use ingested sugars as a direct fuel, just like hummingbirds (Amitai et al., 2010; Voigt & Speakman, 2007; Welch & Suarez, 2008). Additionally, an elevated amount of sugar intake is parallel with an increased speed of paracellular nutrient absorption in fruit bats (Caviedes‐Vidal et al., 2008; Tracy et al., 2007). Therefore, these bats can uptake large quantities of sugar each night. The mechanism by which they use against potential hyperglycemia remains to be uncovered.

Blood glucose is strictly controlled at a constant level by mammals to maintain a normal physiological condition, and the level is normally under 10 mmol/L (Kelm et al., 2011; Kjeld & Ólafsson, 2008; Suh, Paik, & Jacobs, 2007; Umminger, 1975). Kelm et al. (2011) proposed that a higher level of physical activity is a key regulator for blood glucose homeostasis during foraging of nectar‐feeding bats *Glossophaga soricina*. As *Eonycteris spelaea* (nectarivorous) and *Cynopterus sphinx* (frugivorous) of the family Pteropodidae are fruit bats commonly found in Southern China, in this study, these bats were used to explore whether Kelm's hypothesis is also applicable to Pteropodidae bats and to examine our hypotheses: The level of postprandial blood glucose is (1) positively correlated to the amount of sugar intake, but it is (2) negatively correlated to the flight duration.

## 2 MATERIALS AND METHODS

### 2.1 Animals and blood glucose levels upon capture

In the spring and summer (April and June 2015), we captured nonreproductive adult bats using mist nets and hand‐held net in Xishuangbanna, Yunnan Province, Southwest China. Both species of bats were separately kept in a large outdoor cage (3 × 9 × 12 m) shaded by plastic mesh on the edges, and fed with excess fresh fruits purchased from local store or the liquid food containing sugar, honey, and fruit juice according to their food preference. The propatagial vein of bats was punctured with a sterile needle (Soft Lancets 26G; SteriLance, Suzhou, China). The blood glucose level (BL) was analyzed by a glucose meter, Accu‐check Integra (Roche Diagnostics). The BL and body weight (Mb) of all bats were measured immediately upon capture. The average BL and Mb were 4.6 ± 1.62 mmol/L and 48.70 ± 5.21 g for *C. sphinx* (*n *=* *84) and 4.6 ± 1.11 mmol/L and 59.68 ± 9.78 g for *E. spelaea* (*n *=* *115). All experiments began at 1 hr after sunset when the bats started their nocturnal activities, and each bat was used once in a test. To reduce the physical loading of bats, we ended the experiment when the blood glucose levels were relatively stable and then sent them to the habitat.

### 2.2 Blood glucose level in inactive bats after feeding

Oral sucrose tolerance test was conducted for both species of bats (*n *=* *24 for each species). Prior to the experiment, all bats were fasted for at last 12 hr and were randomly divided into four groups (*n *=* *6 in each group). Sucrose that is preferred over glucose by bats was offered during feeding. Each bat was held gently by hand and fed on 20% sucrose in water (w/w) contained in the syringe, and the bat would spontaneously ingest this sugar water. The sucrose dosages used for each group were 1.8 g (group 1), 5.4 g (group 2), and 9 g (group 3)/kg Mb, and the energy provided was approximately equal to 1% to 3% of daily energy expenditure of nectar‐feeding bats as previously described (Kelm et al., 2011). Bat in the control group was not fed. The blood glucose level of bats was measured at fasting stage and also at each 10‐min interval up to 90 min after feeding. Approximately 1.5 μl of whole blood was taken in each measurement, and thus, a total of 15 μl blood sample was used for the entire test of 10 measurements. The bats were rested in cotton bags between measurements.

### 2.3 Blood glucose level in active bats after feeding

The changes of blood glucose level were studied using both species of bats (*n *=* *24 for each species) under two conditions: (1) different time of flight and single feeding and (2) fixed time of flight and multiple feedings.

**Figure 1:**
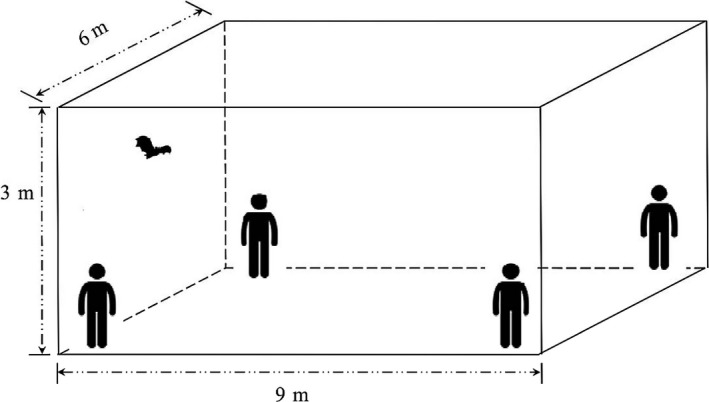
The diagrammatic sketch of flying experiment

In the first condition, we offered once for all bats with sucrose solution 5.4 g/kg Mb that is approximately equal to 2% of daily energy expenditure of nectar‐feeding bats (Kelm et al., 2011). After feeding, these bats were randomly divided into four groups (*n *=* *6 in each group). Bats of control group were individually kept in cotton bags, and those of the rest groups were manually entered into flight test in a 3 × 9 × 12 m plastic tent. Four staffs in the corners of the tent would disturb those cling bats (Figure 1). Flight test continues for a total 40‐min period composed of a 10‐min repeat cycle of flight and rest. The bats were induced to fly for either 20% (8 min, group 1), 50% (20 min, group 2) or 70% (28 min, group 3) of the total time. As described in the previous section, blood glucose levels were firstly determining at fasting state of all bats and then at each 10‐min interval up to 70 min during the experiment.

In the second condition, we selected six bats from each of both phytophagous species. We fed these bats with 2.7 g sucrose/kg Mb every 10 min and encouraged the bats to fly for 2 min after feeding. The energy provided by sugar 2.7 g sucrose/kg Mb is close to 1% of daily energy expenditure of nectar‐feeding bats (Kelm et al., 2011), in which we assume that bats fly 2 min in each 10‐min interval. Bats were kept separately in the cotton bags after flight. Blood glucose level was measured before each hand feeding. The overall experimental period of this test was 90 min.

### 2.4 Blood glucose level in bats under the simulated natural habitat

To simulate the natural feeding conditions of free‐range bats, both species of bats (*n *=* *24 for each species) were randomly divided into four groups (*n *=* *6 for each group) and their blood glucose levels were measured. After grouping, they were fed every 15 min with 5.4 g mixed sugar/kg Mb in a 20% water solution (w/w). The mixed sugar was composed of 26% sucrose, 37% glucose, and 37% fructose. After feeding, these bats were manually entered into flight test in a plastic tent (3 × 9 × 12 m). This test has a total 120‐min period which consists of a 15‐min repeat cycle of flight and rest. The flight and rest cycles for 30%, 45%, 60%, and 75% time groups were 4.5 and 10.5, 6.75 and 8.25, 9 and 6, and 11.25 and 3.75 min, respectively. Blood glucose levels were determining before feeding of all bats and then at each 15‐min interval up to the end of the experiment.

### 2.5 Statistical analysis

We used SPSS 20.0 for Windows to analyze data. All values are given as means ± *SE*, and the *p* value < .05 was considered significant.

## 3 RESULTS

### 3.1 The amount of sucrose intak is correlated positively with blood glucose level in inactive fruit bats

**Figure 2:**
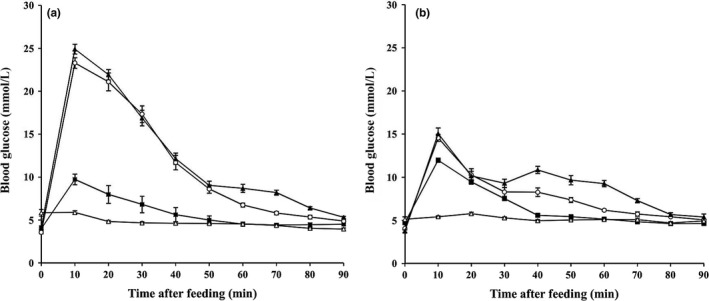
The changes of blood glucose level in resting *Cynopterus sphinx* (a) and *Eonycteris spelaea* (b) fed either 0 (open triangles, control group), 1.8 (filled squares), 5.4 (open circles), or 9 (filled triangles) g of sucrose/kg Mb (*n *=* *6 per group)

At fasting, the mean levels of blood glucose were 4.54 ± 1.73 mmol/L (*C. sphinx*,* n *=* *74) and 4.63 ± 1.11 mmol/L (*E. spelaea*,* n *=* *75). After feeding, the blood glucose concentrations increased rapidly up to their peak values within 10 min (Figure 2). An increase in sucrose intake was significantly correlated with the up regulation of blood glucose level (Spearman's rank, blood glucose peaks: *C. sphinx*,* r*_*s*_ = 0.864, *p *<* *.01, *n *=* *24; *E. spelaea*,* r*_*s*_ = 0.752, *p *<* *.01, *n *=* *23). However, no correlation was seen between the blood glucose levels and time after feeding (Spearman's rank, the time of blood glucose integral: *C. sphinx*,* r*_*s*_ = 0.274, *p *>* *.05, *n *=* *24; *E. spelaea*,* r*_*s*_ = −0.196, *p *>* *.05, *n *=* *23). After ingestion of 9 g of sucrose/kg Mb, the maximum blood glucose concentrations of *C. sphinx* even exceeded 24 mmol/L and the mean value of blood glucose remained at *approx*. 12 mmol/L 90 min after glucose ingestion, whereas in *E. spelaea*, the maximum blood glucose value was remained around 15 mmol/L and the mean value was below 9 mmol/L.

### 3.2 Airborne and continuous fed change the postprandial blood glucose levels in active fruit bats

**Figure 3:**
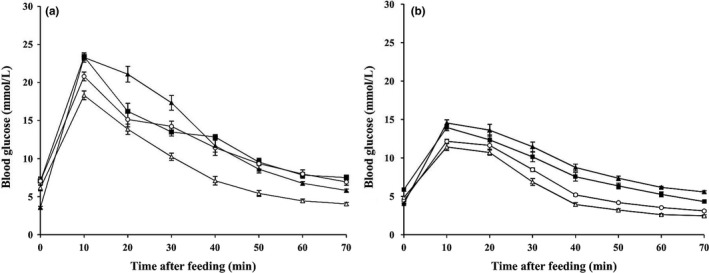
The impact of flight time on blood glucose levels in *Cynopterus sphinx* (a) and *Eonycteris spelaea* (b) after feeding with 5.4 g sucrose/kg Mb. The bats flew for 20% (filled squares), 50% (open circles), 70% (open triangles) of a 10‐min interval or at rest (filled triangles) during the first 40 min (*n *=* *6 per group)

Under the condition of different time of flight and single feeding (Figure 3), we found that the magnitude of the blood glucose spikes showed a negative correlation with the flight time (Pearson's correlation, blood glucose peaks: *C. sphinx*,* r *=* *−0.683, *p *<* *.01, *n *=* *24; *E. spelaea*,* r *=* *−0.504, *p *<* *.05, *n *=* *23). However, the maximum blood glucose levels were reached around 10 min similar to the results of Figure 2, thus no significant correlation between flight activity and time after feeding (Spearman's rank, time of blood glucose elevated: *C. sphinx*,* r*_*s*_ = 0.04 *p *>* *.05, *n *=* *23; *E. spelaea*,* r*_*s*_ = 0.146, *p *>* *.05, *n *=* *23). Compared with resting bats, flying bats had relatively lower magnitude of the blood glucose spikes (Figure 3). For *C. sphinx* at 20 min after feeding, the blood glucose of the group that flew for 70% of the time was significantly lower than that of other groups (one‐way ANOVA: *F*_3, 20_ = 2.97, *p *<* *.05; LSD: *p *<* *.05). For *E. spelaea*, when the bat flew for 50% and 70% of the time, the blood glucose also showed a greater decline than that in the other two groups (one‐way ANOVA: *F*_3, 19_ = 8.07, *p *<* *.01; post hoc LSD: all *p *<* *.05). The postprandial blood glucose levels were negatively correlated with the time of flight.

**Figure 4:**
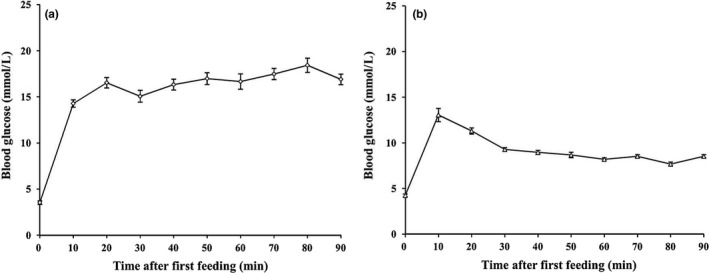
Blood glucose levels in *Cynopterus sphinx* (a) and *Eonycteris spelaea* (b) during feeding 2.7 g of sucrose/kg Mb as a 20% solution every 10‐min interval. After then, the bats were in flight for 20% of time interval (*n *=* *6 per group)

Under the condition of fixed time of flight and multiple feedings, we observed that the blood glucose of *C. sphinx* constantly remained at a high level, reaching an average of 17 mmol/L despite the intermittent low intensive flight activity (Figure 4a). In *E. spelaea*, at 10–20 min after the first feeding, the blood glucose of bats had a rapidly rising stage up to the peak(s) and then fell slightly and kept at approximately 8.6 mmol/L (Figure 4b).

### 3.3 The flight activity change the postprandial blood glucose level under the simulated natural habitat

**Figure 5:**
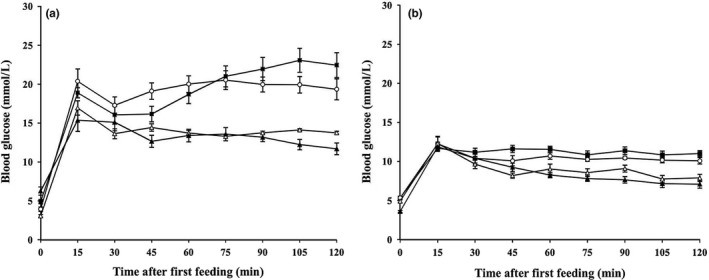
Blood glucose level changes in *Cynopterus sphinx* (a) and *Eonycteris spelaea* (b) during feeding 5.4 g sugar/kg Mb as a 20% sugar solution every 15‐min interval. The sugar consisted of 37% glucose, 37% fructose, and 26% sucrose. After then, the animals were in flight for 30% (filled squares), 45% (open circles), 60% (open triangles) or 75% (filled triangles) of time intervals (*n *=* *6 per group)

Every 15 min, we fed the bats a food that simulated the natural diet and then subjected the bats to fly different times in intervals. After 15 min postfeeding, the blood glucose levels were gradually decline and then tended to be stabilized (Figure 5). When the bat spent more time to fly, the blood glucose would fall off and soon maintain at a certain level. Over the last 60 min, it showed a negative correlation between the mean blood glucose levels and flight time (Pearson's correlation: *C. sphinx*,* r* = −0.57 *p *<* *.05, *n *=* *23; *E. spelaea*,* r* = −0.59, *p *<* *.01, *n *=* *22). In *E. spelaea*, only when the bats spent at least 60% of their time in the air could the mean blood glucose levels consistently reach values below 8 mmol/L. However, in *C. sphinx*, when the bats spent 60%–75% of the time in flight, the mean blood glucose levels did not decline to the expected values. Nonetheless, the mean blood glucose levels of the groups spending 60%–75% of the time in the air were distinctly lower than those of other two groups.

## 4 DISCUSSION

In this study, we found that *C. sphinx* and *E. spelaea* showed highly variable blood glucose levels after ingestion of different quantity of sucrose. The bats rested directly after feeding experienced a period of high levels of blood glucose. Notably, *C. sphinx* exhibited an extreme blood glucose peak exceed 24 mmol/L after feeding but the fasting value was *approx*. 4.54 ± 1.73 mmol/L. The amount of sugar intake and flight intensity influenced significantly the magnitude of the blood glucose spikes and the duration of the high blood glucose levels. When the blood glucose had reached a high level, *E. spelaea* was able to reduce it to their fasting values through sustained, high intensive flight, such as flight for approximately 60%–75% of the time. This regulation of blood glucose is commonly found in mammals (Kjeld & Ólafsson, 2008; Umminger, 1975). Although *C. sphinx* did not regulate their blood glucose levels to their fasting values, their blood glucose levels largely dropped when they spent 60%–75% time to fly. This implies that both *C. sphinx* and *E. spelaea* may not merely regulate their postfeeding blood glucose levels by insulin‐triggered glucose regulatory system. Our results were similar to those of Kelm et al. (2011) who conducted blood glucose research in *G. soricina*.

Among mammals, the strict and accurate blood glucose control is vital because abnormal blood glucose levels can cause serious damage to health (Brownlee, 2001; Ceriello, 2003; Nathan, 1996; Rolo & Palmeira, 2006; Suh et al., 2007). However, our findings in this study are uncommon. In general, blood glucose regulation leads to health benefits and the floor level and upper limit of blood glucose should not be affected by dietary uptake or the degree of energy consumption (Kjeld & Ólafsson, 2008; Suh et al., 2007; Umminger, 1975). Some researches indicate that a negative correlation exists between body mass and blood glucose level in certain species, but the level of postprandial blood glucose over 10 mmol/L in *C. sphinx* and *E. spelaea* are unusual in small mammals (Braun & Sweazea, 2008; Kjeld & Ólafsson, 2008; Umminger, 1975). Furthermore, the values of blood glucose in *C. sphinx* reached over 24 mmol/L after feeding. This level was extremely higher than any records of mammals (Helversen & Reyer, 1984; Voigt, Kelm & Visser 2006) and would be considered subhealth in humans and other mammals (Gavin, Alberti, Davidson, & DeFronzo, 1997; Kjeld & Ólafsson, 2008; Umminger, 1975).

We considered two scenarios to explain these findings. First, despite having blood glucose levels that are above the mammalian norm, fruits bats (*C. sphinx* and *E. spelaea*) have evolved some mechanisms which could rapidly eliminate the possible occurrence of hyperglycemia, and thus fruit bats (*C. sphinx* and *E. spelaea*) can endure high blood glucose levels. Another scenario is that high‐intensive flight could balance the postprandial blood glucose in nature (see below for details).

Assuming that fruit bats (*C. sphinx* and *E. spelaea*) are able to bear the high blood glucose levels, there must be some special, currently unknown mechanisms to deal with all kind of side effect of high levels of blood glucose. Some researchers have pointed that high blood glucose typically increases the formation of unhealthful products in body, and one of typical is glucated hemoglobin (HbA1c), a toxic advanced glycogen end‐products (AGE) (Kilpatrick, 2000; Nathan et al., 2008). The extent of hemoglobin glycation correlates strongly with the level of ambient glycemia during several weeks and HbA1c plays a crucial role in cardiovascular disease and diabetes (Brownlee, 2001; Brunner, Schvartz, Priego‐Capote, Couté, & Sanchez, 2009; Ceriello, 2003; Kawahito, Kitahata, & Oshita, 2009; Nathan, 1996; Suh et al., 2007). However, Kelm's test for HbA1c clarified further that high blood glucose levels in fruit bats do not last very long (Kelm et al., 2011).

High levels of blood glucose and abnormal blood glucose fluctuations may also increase the number of reactive oxygen species (ROS) (Brownlee, 2001; Buffenstein, Edrey, Yang, & Mele, 2008; Choi, Benzie, Ma, Strain, & Hannigan, 2008; Kawahito et al., 2009). In general, when the level of ROS exceed the normal, it may damage proteins and lipids, even DNA, and it also affects the animal aging and leads to metabolic syndrome and diabetes because of ROS‐induced oxidative stress (Barja, 2002; Brunet‐Rossinni & Austad, 2004; Buffenstein et al., 2008). ROS can also lead to pancreatic β‐cell dysfunction, tissues insulin resistance, and declining insulin production (Brownlee, 2001; Brunner et al., 2009; Kawahito et al., 2009; Vincent, Russell, Low, & Feldman, 2004). Follow this point, fruit bats must have the ability to prevent excess production of ROS or reduce oxidative stress raised by a huge fluctuation of blood glucose (Brunner et al., 2009; Buffenstein et al., 2008; Kawahito et al., 2009; Munshi‐South & Wilkinson, 2010; Vincent et al., 2004). However, other researchers hold different opinions. They thought that there is no unequivocal support that ROS production would give rise to oxidative damage and shorter lifespan. ROS productions are similar between long‐lived and short‐lived mammals, and some long‐lived species even have a higher level of ROS production (Brunet‐Rossinni & Austad, 2004; Buffenstein et al., 2008; Yin et al., 2016).

Kelm et al. (2011) suggested that tolerating hyperglycemia might enable bats to use the blood glucose to fuel high rates of glycolysis in the muscle cells during flight in a certain degree and high rates of glycolysis might serve to reduce oxidative stress and control ROS formation (Kelm et al., 2011).

Glycogenolysis supplies 70%–80% of the glucose needs during physical exercise in most mammals. For example, exogenous fuel by dietary ingestion maximally supports 30% of the metabolic requirements in exercising humans (Jeukendrup & Jentjens, 2000; Roberts, Weber, Hoppeler, Weibel, & Taylor, 1996). Some authors considered that the speed of intestinal glucose absorption and cellular glucose transport would limit the utilization rate of exogenous fuel (Hayashi, Wojtaszewski, & Goodyear, 1997; Jeukendrup & Jentjens, 2000). However, recent studies have shown that both nectar‐feeding and fruit‐feeding bats could fuel directly with exogenous substrate to meet almost entirely demands of energy metabolism both at rest and flight (Voigt & Speakman, 2007; Welch & Suarez, 2008). This finding implies that the fruit bats are capable of supplying their extremely high energetic needs during flight with recently ingested sufficient sugars. In fruit bats, intestinal sucrose concentrations are high and sugar uptake is quick (Hernandez & del Rio, 1992; Voigt & Speakman, 2007; Welch & Suarez, 2008). Therefore, intestinal carbohydrate uptake cannot be a limiting factor. In addition, in fruit bats, as in hummingbirds, except the active transport, an additional pathway of sugar ingestion is called paracellular pathway that can accelerate carbohydrate absorption (Caviedes‐Vidal et al., 2007; Tracy et al., 2007). It has been found that the signaling pathway triggered by insulin or muscle contraction increase cellular glucose uptake (Kelm et al., 2011). Some researchers have mentioned that these factors may have additive effects on glucose transporter protein translocation to speed up glucose uptake (Bryant, Govers, & James, 2002; Hayashi et al., 1997; Holloszy, 2003).

When we induced bats to a low intensive of flight during multiple feedings, their blood glucose levels did not quickly dropped. It is possible that the active enzymes of glycogenesis are saturated or that other regulatory mechanisms do not keep up with intestinal glucose absorption and thereby fruit bats cannot remove persistently high levels of glucose from the blood stream. Instead, it seems that blood glucose levels are positively correlated with the amount of glucose intake.

Regardless of fruit bats's ability to endure high blood glucose levels, our results showed that the flight activity of fruit bats clearly serves to reduce the peak of blood glucose levels and the duration of hyperglycemia. For both *C. sphinx* and *E. spelaea*, when they spent more time to fly (e.g., 60%–75% of their time), the average levels of blood glucose were lower than those bats flew less and quickly stabilized. In the field, the foraging behavior of fruit bats will last for a long time every night, even up to 12 hr, and these foraging events are alternated with short‐times rest (Winter & von Helversen, 1998). As *Glossophaga commissarisi* is active for an average of 60% and a maximum of 80% of its time at night in nature (Rothenwöhrer, Becker, & Tschapka, 2011), our results suggest that this level of activity may serve to prevent fruit bats from high blood glucose levels after feeding.

High activity may decline blood glucose levels, and it can support that the high‐sugar diet in fruit bat and high‐sugar diet allows bats to allocate more energy to other beneficial activities, such as searching for new food sources or habitat, increasing the frequency of mating or other social interactions (Kelm et al., 2011).

Concurrently, the food in natural habitats may be spatially scattered, which, considering the competition of food resource in intraspecific and interspecific, might increase the foraging costs for bats by requiring more flight time and the travelling of greater distances. However, flight more and travelling longer contribute to pollen and seed dispersal of foraging fruit bats. In certain seasons, plants produce vast amounts of food for bats to ingest, only flight activity would prevent extreme blood glucose levels occur to bats. In this situation, fruit bats could allocate redundant energy to other activities (such as searching for habitat, mating behavior and other social interactions), which would be beneficial to the continuation of their genes and populations. Recent behavioral observations of fruit bats in captivity may help to confirm this hypothesis. Beyond the requirement for foraging, captive fruit bats continue to engage in other flight activities.

We noted that some differences between the two species in blood glucose level over time in the various tests, such as blood glucose levels of *E. spelaea,* raised to only half that of *C. sphinx,* and there was a jump of blood glucose level in *C. sphinx* between feeding 1.8 and 5.4 g sucrose/kg Mb, while *E. spelaea* did not. It may be related to their ecology, for instance, different habitat environment, living habit, and foraging strategy.

*Eonycteris spelaea* uses nectar as its main food source, whereas *C. sphinx* mainly feeds on fruits. The mean sugar concentration of neotropical bat flowers is 17% (w/w), and maximum sugar concentration in fruits and nectar may reach 30% (Baker et al., 1998; Helversen & Reyer, 1984). However, fruiting plants provide energy rewards that exceed nectar rewards at flowers of bats‐pollinated plants. *C. sphinx*, the arboreal bats, generally are dispersed into small group or live alone and have lower intraspecific competition pressure, and often perch near orchard and rich food resources. While *E. spelaea*, generally formed a huge colony in a cave and thus exacerbate the intraspecific competition pressure, and their roosts are far apart from foraging sites. It is reported that the foraging distance of nectar‐feeding bats at night usually up to 20–40 km (Fenton, 2001). Therefore, foraging costs per energy reward unit of *E. spelaea* would far exceed those of *C. sphinx*. Based on this, *E. spelaea* may need to obtain more sugar each night to sustain its daily energy requirements. Because of ingesting more sugar at the wild, *E. spelaea*, perhaps, are better than *C. sphinx* in blood glucose regulation.

In conclusion, high intensive of flight is a guarantee of fruit bats to maintain the high‐sugar diet. High‐intensive flight may serve to individual foraging success and community interaction. For some plants, fruit bats are pollinator and seed spreaders, and high‐intensive flight would significantly enhance the efficiency of pollination and seed diffusion.

## CONFLICT OF INTEREST

None declared.

## AUTHOR CONTRIBUTION

Xingwen Peng, Libiao Zhang, and Zhixiao Liu designed the study. Xingwen Peng, Libiao Zhang, Xiangyang He, Qi Liu, Hui Liu, and Qin Zhang performed the experiments. Xingwen Peng, Libiao Zhang, Xiangyang He, and Qi Liu collected the specimens. Xingwen Peng, Libiao Zhang, Zhixiao Liu, Yunxiao Sun, Jie Liang, and Zhen Peng analyzed the data and wrote the manuscript.
